# Supplementary material for: Association between C-Maf-inducing protein gene rs2287112 polymorphism and schizophrenia
Source: PeerJ. 2021 Aug 20;9:e11907. doi: 10.7717/peerj.11907 (PMC8381876; doi:10.7717/peerj.11907)
Supplement: Supplemental Information 2 [file peerj-09-11907-s002.doc]

Sex: 1=male, 2=female

Group: 1=case, 0=control
