# Supplementary material for: Association between C-Maf-inducing protein gene rs2287112 polymorphism and schizophrenia
Source: PeerJ. 2021 Aug 20;9:e11907. doi: 10.7717/peerj.11907 (PMC8381876; doi:10.7717/peerj.11907)
Supplement: Supplemental Information 3 [file peerj-09-11907-s003.doc]

| SNPs |  | Genotype | Case(n) | Control(n) | *χ^2^* | *P* | *Padj* | OR(95CI) |
| --- | --- | --- | --- | --- | --- | --- | --- | --- |
| Dominant | Total | AA | 605 | 617 | 0.869 | 0.351 | 0.908 | 1 |
|  |  | TA+TT | 135 | 155 |  |  |  | 0.885(0.685-1.144) |
|  | Male | AA | 354 | 341 | 1.079 | 0.299 | 0.908 | 1 |
|  |  | TA+TT | 80 | 92 |  |  |  | 0.838(0.599-1.170) |
|  | Female | AA | 251 | 276 | 0.04 | 0.841 | 0.908 | 1 |
|  |  | TA+TT | 55 | 63 |  |  |  | 0.960(0.643-1.432) |
| Codominant | Total | AA | 605 | 617 | 1.115 | 0.573 | 0.908 | 1 |
|  |  | TA | 128 | 145 |  |  |  | 0.898(0.690-1.168) |
|  |  | TT | 7 | 10 |  |  |  | 0.697(0.263-1.845) |
|  | Male | AA | 354 | 341 | 1.161 | 0.56 | 0.908 | 1 |
|  |  | TA | 74 | 84 |  |  |  | 0.849(0.600-1.199) |
|  |  | TT | 6 | 8 |  |  |  | 0.722(0.248-2.104) |
|  | Female | AA | 251 | 276 | 0.258 | 0.879 | 0.908 | 1 |
|  |  | TA | 54 | 61 |  |  |  | 0.973(0.650-1.458) |
|  |  | TT | 1 | 2 |  |  |  | 0.550(0.050-6.100) |
| Overdominant | Total | AA+TT | 612 | 627 | 0.585 | 0.444 | 0.908 | 1 |
|  |  | TA | 128 | 145 |  |  |  | 0.903(0.694-1.174) |
|  | Male | AA+TT | 360 | 349 | 0.246 | 0.62 | 0.908 | 1 |
|  |  | TA | 74 | 84 |  |  |  | 0.912(0.633-1.314) |
|  | Female | AA+TT | 252 | 278 | 0.013 | 0.908 | 0.908 | 1 |
|  |  | TA | 54 | 61 |  |  |  | 0.977(0.652-1.463) |

Stable 1 Genotypic distributions of rs77700579 between SCZ patients and healthy with different genetic models

*P*_adj_ represent *P* corrected by FDR, *OR* is abbreviation of Odds ratio, *95%CI* is abbreviation of 95% confidence interval.
