# Supplementary material for: Association between C-Maf-inducing protein gene rs2287112 polymorphism and schizophrenia
Source: PeerJ. 2021 Aug 20;9:e11907. doi: 10.7717/peerj.11907 (PMC8381876; doi:10.7717/peerj.11907)
Supplement: Supplemental Information 4 [file peerj-09-11907-s004.doc]

Stable 2 Genotypic distributions of rs12925980 between SCZ patients and healthy with different genetic models

| SNPs |  | Genotype | Case(n) | Control(n) | *χ^2^* | *P* | *Padj* | *OR*(95CI) |
| --- | --- | --- | --- | --- | --- | --- | --- | --- |
| Dominant | Total | TT | 127 | 122 | 0.716 | 0.398 | 0.907 | 1 |
|  |  | CT+CC | 602 | 650 |  |  |  | 0.889(0.677-1.167) |
|  | Male | TT | 66 | 76 | 0.928 | 0.335 | 0.907 | 1 |
|  |  | CT+CC | 367 | 354 |  |  |  | 0.838(0.584-1.201) |
|  | Female | TT | 51 | 56 | 0.033 | 0.856 | 0.907 | 1 |
|  |  | CT+CC | 248 | 283 |  |  |  | 0.962(0.635-1.459) |
| Codominant | Total | TT | 127 | 122 | 0.746 | 0.689 | 0.907 | 1 |
|  |  | CT | 352 | 377 |  |  |  | 0.897(0.672-1.196) |
|  |  | CC | 250 | 273 |  |  |  | 0.879(0.650-1.189) |
|  | Male | TT | 76 | 66 | 0.941 | 0.625 | 0.907 | 1 |
|  |  | CT | 204 | 213 |  |  |  | 0.832(0.568-1.218) |
|  |  | CC | 150 | 154 |  |  |  | 0.846(0.567-1.261) |
|  | Female | TT | 51 | 56 | 0.195 | 0.907 | 0.907 | 1 |
|  |  | CT | 148 | 164 |  |  |  | 0.991(0.638-1.538) |
|  |  | CC | 100 | 119 |  |  |  | 0.923(0.581-1.466) |
| Overdominant | Total | TT+CC | 377 | 395 | 0.044 | 0.833 | 0.907 | 1 |
|  |  | CT | 352 | 377 |  |  |  | 0.978(0.799-1.198) |
|  | Male | TT+CC | 226 | 220 | 0.265 | 0.607 | 0.907 | 1 |
|  |  | CT | 204 | 213 |  |  |  | 1.00 (0.74-1.34) |
|  | Female | TT+CC | 151 | 175 | 0.08 | 0.777 | 0.907 | 1 |
|  |  | CT | 148 | 164 |  |  |  | 1.07 (0.76-1.52) |

*P*_adj_ represent *P* corrected by FDR, *OR* is abbreviation of Odds ratio, *95%CI* is abbreviation of 95% confidence interval
