# Supplementary material for: Association between C-Maf-inducing protein gene rs2287112 polymorphism and schizophrenia
Source: PeerJ. 2021 Aug 20;9:e11907. doi: 10.7717/peerj.11907 (PMC8381876; doi:10.7717/peerj.11907)
Supplement: Supplemental Information 5 [file peerj-09-11907-s005.doc]

Stable 3 Genotypic distributions of rs3751859 between SCZ patients and healthy with different genetic models

| SNPs |  | Genotype | Case(n) | Control(n) | *χ^2^* | *P* | *Padj* | *OR*(95CI) |
| --- | --- | --- | --- | --- | --- | --- | --- | --- |
| Dominant | Total | TT | 532 | 585 | 0.975 | 0.323 | 0.734 | 1 |
|  |  | GT+GG | 174 | 187 |  |  |  | 1.108(0.904-1.358) |
|  | Male | TT | 320 | 334 | 0.041 | 0.84 | 0.911 | 1 |
|  |  | GT+GG | 99 | 100 |  |  |  | 1.033 (0.752-1.419) |
|  | Female | TT | 212 | 251 | 0.012 | 0.911 | 0.911 | 1 |
|  |  | GT+GG | 75 | 87 |  |  |  | 1.021 (0.713-1.461) |
| Codominant | Total | TT | 532 | 585 | 5.984 | 0.05 | 0.225 | 1 |
|  |  | GT | 150 | 176 |  |  |  | 0.941(0.735-1.206) |
|  |  | GG | 24 | 11 |  |  |  | 2.386(1.157-4.920) |
|  | Male | TT | 320 | 334 | 1.429 | 0.489 | 0.734 | 1 |
|  |  | GT | 86 | 92 |  |  |  | 0.976 (0.700-1.359) |
|  |  | GG | 13 | 8 |  |  |  | 1.696 (0.694-4.147) |
|  | Female | TT | 212 | 251 | 6.441 | 0.04* | 0.225 | 1 |
|  |  | GT | 64 | 84 |  |  |  | 0902 (0.621-1.310) |
|  |  | GG | 11 | 3 |  |  |  | 4.341 (1.195-15.765) |
| Overdominant | Total | TT+GG | 556 | 596 | 0.516 | 0.472 | 0.734 | 1 |
|  |  | GT | 150 | 176 |  |  |  | 0.918(0.717-1.175) |
|  | Male | TT+GG | 333 | 342 | 0.058 | 0.809 | 0.911 | 1 |
|  |  | GT | 86 | 92 |  |  |  | 0.960(0.690-1.336) |
|  | Female | TT+GG | 223 | 254 | 0.56 | 0.454 | 0.734 | 1 |
|  |  | GT | 64 | 84 |  |  |  | 0.868 (0.598-1.258) |

*P*_adj_ represent *P* corrected by FDR, *OR* is abbreviation of Odds ratio, *95%CI* is abbreviation of 95% confidence interval.
