# Supplementary material for: Association between C-Maf-inducing protein gene rs2287112 polymorphism and schizophrenia
Source: PeerJ. 2021 Aug 20;9:e11907. doi: 10.7717/peerj.11907 (PMC8381876; doi:10.7717/peerj.11907)
Supplement: Supplemental Information 6 [file peerj-09-11907-s006.doc]

Stable 4 Genotypic distributions of rs2287112 between SCZ patients and healthy with different genetic models

| SNPs |  | Genotype | Case(n) | Control(n) | *χ^2^* | *P* | *Padj* | *OR*(95CI) |
| --- | --- | --- | --- | --- | --- | --- | --- | --- |
| Dominant | Total | GG | 326 | 365 | 0.975 | 0.323 | 0.563 | 1 |
|  |  | GA+AA | 402 | 407 |  |  |  | 1.108(0.904-1.358) |
|  | Male | GG | 200 | 204 | 0.044 | 0.834 | 0.834 | 1 |
|  |  | GA+AA | 231 | 229 |  |  |  | 1.029(0.788-1.344) |
|  | Female | GG | 126 | 161 | 1.642 | 0.2 | 0.563 | 1 |
|  |  | GA+AA | 171 | 178 |  |  |  | 1.228(0.897-1.680) |
| Codominant | Total | GG | 326 | 365 | 1.961 | 0.375 | 0.563 | 1 |
|  |  | GA | 327 | 320 |  |  |  | 1.147(0.926-1.422) |
|  |  | AA | 75 | 87 |  |  |  | 0.963(0.683-1.358) |
|  | Male | GG | 200 | 204 | 0.432 | 0.806 | 0.834 | 1 |
|  |  | GA | 186 | 179 |  |  |  | 1.060(0.799-1.407) |
|  |  | AA | 45 | 50 |  |  |  | 0.918(0.587-1.436) |
|  | Female | GG | 126 | 161 | 2.236 | 0.327 | 0.563 | 1 |
|  |  | GA | 141 | 141 |  |  |  | 1.278(0.919-1.777) |
|  |  | AA | 30 | 37 |  |  |  | 1.036(0.607-1.769) |
| Overdominant | Total | GG+AA | 401 | 452 | 1.915 | 0.166 | 0.563 | 1 |
|  |  | GA | 327 | 320 |  |  |  | 1.156(0.942-1.418) |
|  | Male | GG+AA | 245 | 254 | 0.292 | 0.589 | 0.757 | 1 |
|  |  | GA | 186 | 179 |  |  |  | 1.077(0.822-1.411) |
|  | Female | GG+AA | 156 | 198 | 2.219 | 0.136 | 0.563 | 1 |
|  |  | GA | 141 | 141 |  |  |  | 1.269(0.927-1.737) |

*P*_adj_ represent *P* corrected by FDR,* represent *P*< 0.05, *OR* is abbreviation of Odds ratio, *95%CI* is abbreviation of 95% confidence interval.
